# Optimization of machining parameters for turning operation of heat-treated Ti-6Al-3Mo-2Nb-2Sn-2Zr-1.5Cr alloy by Taguchi method

**DOI:** 10.1038/s41598-024-65786-8

**Published:** 2024-07-17

**Authors:** Ramadan N. Elshaer, Ali Abd El-Aty, Esraa M. Sayed, Azza F. Barakat, Arafa S. Sobh

**Affiliations:** 1 Tabbin Institute for Metallurgical Studies, Cairo, Egypt; 2 Mechanical Engineering Department, Faculty of Engineering, Helwan University, Cairo, Egypt; 3 Mechanical Engineering Department, College of Engineering et al Kharj, Prince Sattam Bin Abdulaziz University, 11942 Al Kharj, Saudi Arabia; 4 School of Engineering and Applied Sciences, Nile University, Giza, Egypt

**Keywords:** Heat treatment, Turning parameters, TC21, Wear of tool insert, Surface roughness, Engineering, Materials science

## Abstract

TC21 alloy is a high-strength titanium alloy that has been gaining attention in various industries for its excellent combination of strength, toughness, and corrosion resistance. Given that this alloy is hard to cut material, therefore this study aims to optimize the process parameters of Turing this alloy under different conditions (i.e. as-received alloy, and heat-treated alloy). The L9 Taguchi approach-base orthogonal array is used to determine the optimum cutting parameters and the least number of experimental trials required. The achievement of this target, three different cutting parameters are used in the experimental work; each cutting parameter has three levels. The cutting speeds are chosen as 120, 100, and 80 m/min. The feed rates’ values are 0.15, 0.1, and 0.05, mm/rev, and the depth of cut values are 0.6, 0.4, and 0.2 mm. After applying three steps of heat treatment (First step: is heating the sample to 920 °C for 1 h then decreasing to 820 °C also for 1 h, second step: cooling the sample to room temperature by water quenching (WQ), the third step: holding the sample at 600 °C for 4 h (Aging process)). The results revealed that the triple heat treatment led to the change in the microstructure from (α + β) to (α + β) with secondary α platelets (α_s_) formed in residual β matrix leading to a decreased surface roughness by 56.25% and tool wear by 24.18%. The two most critical factors that affect the tool insert wear and surface roughness are the death of cut and cutting speed, which contribute 46.6% and 46.7% of the total, respectively. Feed rate, on the other hand, has the least importance, contributing 20.2% and 31.9% respectively.

## Introduction

Nowadays, Titanium alloys are significant in a variety of domains and uses. Because of their high ratio of strength to weight, titanium alloys have a significant contribution, especially in the trials in biomedical, aerospace, automotive, and communication engineering. Titanium alloys have a series of grades such as Ti555.3, Ti6Al4V, and their new versions like TC21 alloy^1–3^.

The TC21 alloy represents a new version of titanium alloys, characterized by its chemical composition of Ti-6Al-3Mo-2Zr-2Sn-2Nb-1.5Cr-0.1Si. This alloy features a dual-phase (α + β) microstructure, making it versatile for various applications, either as-received or heat-treated^4^. It exhibits high tensile strength and toughness, even at elevated temperatures, and despite its lightweight, it offers substantial corrosion resistance^5^. Although TC21 is a promising material for diverse applications including military, automotive, aerospace, biomedical devices, sports cars, and high-end sporting goods and consumer electronics, its implementation is hindered by high raw material costs and its challenging machinability^6^. These factors contribute to poor surface quality, reduced machining efficiency, and accelerated tool wear^7^. Consequently, there is a significant need for in-depth research into the machining processes for this alloy. Previous studies have explored the turning process of TC21 Ti-alloy, highlighting these challenges^5–7^.

Over the last few decades, the determination of cutting parameters has been a major challenge, traditionally addressed using full factorial methods, which are both costly and time-consuming^8^. Recent advancements in parameter selection techniques have led to the development of more efficient methods to identify optimal machining parameters^9^. Among these methods are ANOVA, Taguchi, Artificial Neural Network (ANN), and Response Surface Methodology (RSM)^10–15^. As new titanium alloys like TC21 Ti-alloy are developed, it becomes crucial to study their performance in various applications. This includes examining how the TC21 Ti-alloy behaves in metal forming, how cutting parameters and microstructure are affected by heat treatment, and its overall machinability, particularly in processes such as turning, milling, and turn-milling, where machinability remains a significant challenge^11,12^.

The mechanical properties of the TC21 Ti-alloy surpass those of Ti6Al4V and Ti555.3 alloys, leading to its application in various critical fields. Nevertheless, the primary challenge with this alloy is its difficult machinability^13^. Numerous studies have focused on this issue, employing a range of machining parameters to conduct turning operations^10–16^. These studies have predominantly optimized the turning cutting conditions using the Taguchi method. Additionally, some researchers have utilized the full factorial technique along with the fractional factorial approach of the Taguchi method^16^. The findings of these investigations indicate that the Taguchi method, compared to the full factorial method, is more suitable for evaluating the machinability of materials that are difficult to cut.

Isothermal tensile tests were used in various investigations to analyze the deformation behaviour of the TC21 alloy, using stress–strain curves to determine that flow stress inversely correlates with temperature, while maintaining a direct relationship with strain rate^17–22^. In parallel, other studies employed constitutive modeling and high-temperature tensile tests under hot deformation conditions, identifying a direct link between strain and stress, and an inverse relationship between flow stress and strain rate^18^. Additional research focused on the effects of tool microstructure and variations in tool rake angle on the machinability of TC21 Ti-alloy^19^. Using finite element analysis, these studies assessed the cutting performance of the alloy, while turning operations were simulated using the Johnson–Cook (JC) technique and 3D finite element analysis^20^. The findings indicated that adjustments to the tool rake angle decreased scrape serration, reduced wear on the tool insert, and enhanced the machining efficiency of the alloy. However, it is noted that 3D simulations are highly reliant on experimental data, indicating certain limitations in their application.

In addition to turning studies, various milling studies utilized three distinct methods to investigate the cutting forces involved^23–26^. These studies analyzed the impact of cutting forces, the temperature during cuts, and the lifespan of milling cutters in relation to milling parameters, wear of the milling cutter, and the material of the tool^23^. Results indicated that cutting forces, the degree of cutting heat, and the life of the cutter significantly affect the tool material, wear, and milling parameters^24^. Concurrently, some researchers focused exclusively on how milling parameters influenced cutting forces, temperatures, and tool life^25^. Additionally, under different machining conditions, the wear on titanium, aluminum, and nitrogen-titanium-nitride-coated carbide tools, as well as the deposition from physical vapor, were examined^26^. The findings highlighted a strong relationship between increases in tool wear and the magnitude of the cutting force increment.

The Response Surface Methodology, along with the Taguchi technique, has been used to optimize cutting conditions. This methodology helped in developing models to predict surface roughness, demonstrating that the primary objective of optimization is to maximize the material removal rate. Additionally, a combination of response surface methodology and genetic algorithms was utilized to fine-tune the machining parameters. Surface roughness values were determined by averaging measurements from three different areas on the workpiece’s surface^27^.

In this study, the Taguchi method was applied using Minitab 19 to optimize turning parameters and identify ideal conditions that minimize surface roughness and tool insert wear. Subsequently, the ANOVA method was used to pinpoint the most critical parameters. Given the demanding machining requirements of the TC21 Ti-alloy, a tungsten carbide-coated cutting tool (DCMT 11 T 304-NF NS 4125) was employed. The machining process was conducted twice to ensure reliability in the results, and the average of the two responses was calculated.

## Material and method

### Material description, heat treatment stages, experimental equipment, cutting tool, and measuring tools

**Figure 1:**
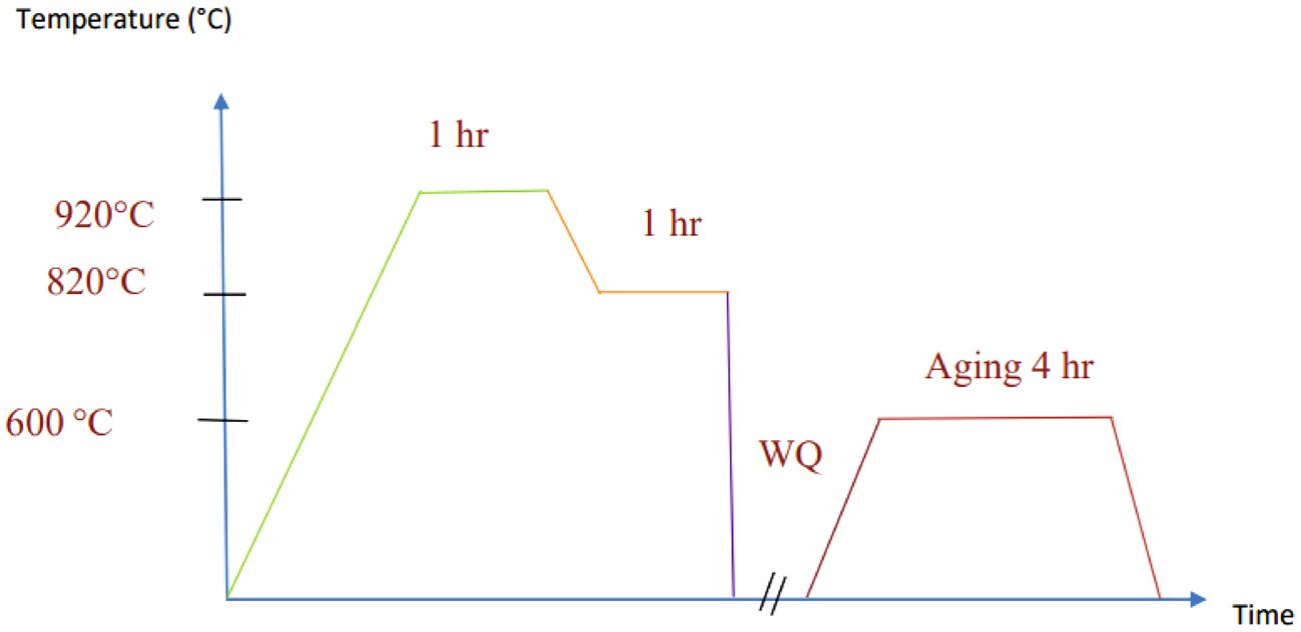
Heat treatment cycle.

**Figure 2:**
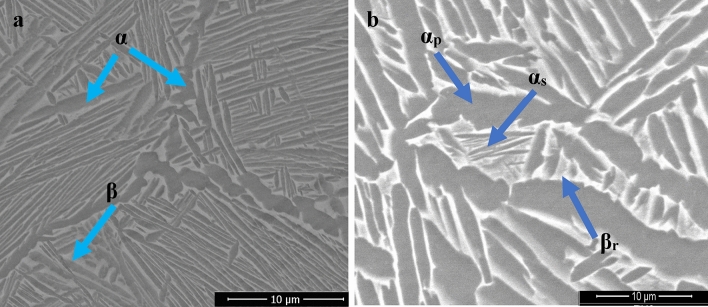
The Microstructure of (**a**) initial and (**b**) heat-treated alloy.

The materials used in this study is TC21 Ti-alloy, which has a microstructure made up of two phases (α + β) and a chemical composition of Ti—6Al—3Mo—2Nb—2Sn—2Zr—1.5Cr as received^28^. The as-received sample is treated in three stages: first, it is heated to 920 °C for an hour and then cooled to 820 °C for an hour; second, water quenching (WQ) is used to cool it to room temperature; and third, it is aged for four hours at 600 °C as shown in Fig. 1. This triple heat treatment transformed the microstructure of the TC21 alloy from an (α + β) structure to a new (α + β) structure with the formation of secondary α platelets (αs) within the remaining β matrix as shown in Fig. 2. The transformation of the microstructure from (α + β) to (α + β) with αs platelets is believed to be responsible for the observed enhancements in hardness and strength of the material. Therefore, there were an increase in the material's hardness and strength: the hardness of the heat-treated TC21 alloy increased significantly, rising 44.63% to 50.33 HRC, and the heat treatment improved the TC21 alloy's overall strength in addition to increasing its hardness.

**Figure 3:**
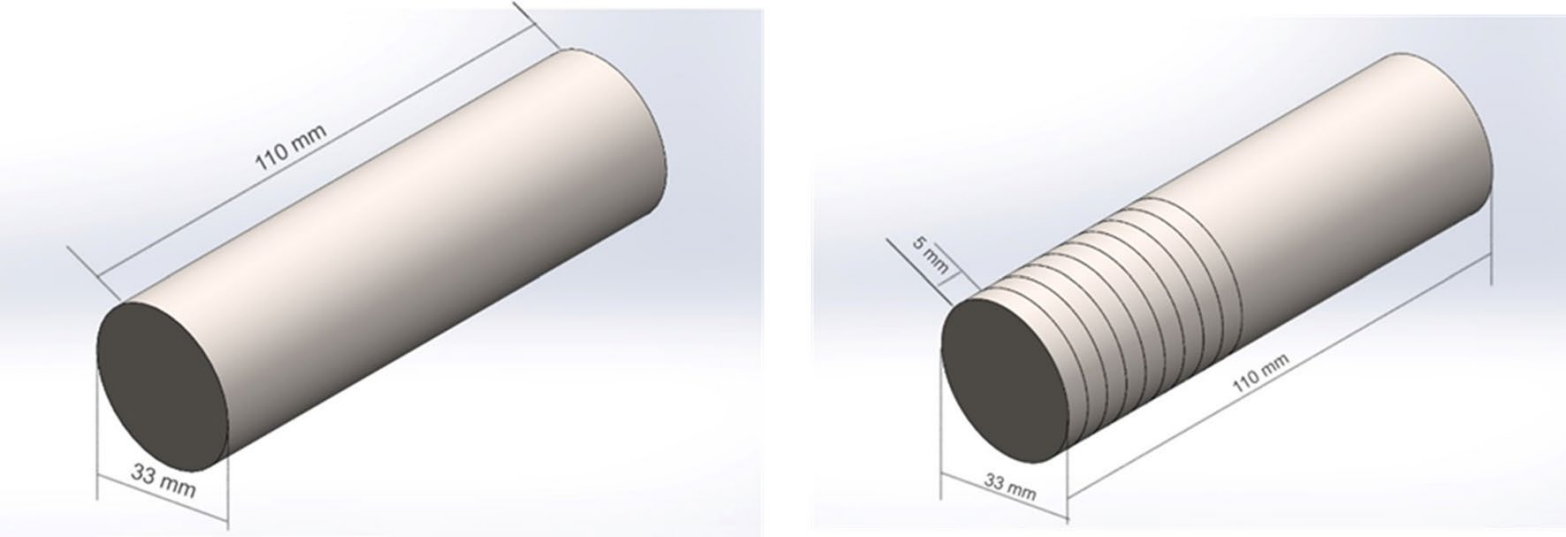
Work sample.

Turning experiments were carried out on CNC machine with a maximum spindle speed of 3000 rpm, known as GOOD WAY to machine the TC21 alloy sample, which had a length of 110 mm and a diameter of 33 mm. The machining process involved conducting 9 separate experiments, with each experiment performed on a 5 mm length section along the sample's total length as shown in Fig. 3. The three key experimental parameters are cutting speed, feed rate, and cutting depth. The surface roughness of the TC21 Ti-alloy specimen was assessed using the DIAVITE DH-5 surface roughness tester, with the surface roughness value (Ra) representing the average taken from three different surface locations. Due to the challenging nature of machining materials like TC21, indexable tungsten-coated carbide cutting tools (DCMT 11 T 304-NF NS 4125) with a 0.4 mm nose radius are employed. Additionally, tool wear for these inserts was examined using scanning electron microscopy (SEM).

### Experimental design via the Taguchi approach

The Taguchi method utilizes a fractional factorial design to decrease the total number of experiments needed. An orthogonal array L9 was used to conduct experiments for three parameters (feed rate, cutting depth, and speed of cut), where each tested at three distinct levels (levels 1, 2, and 3). The specific levels and the layout of the orthogonal array for these parameters are detailed in Tables 1 and 2.

**Table 1:** Parameters and Their Corresponding Levels for the Study.

Factors	**Level** (1)	**Level (2)**	**Level (3)**
Speed of cut V(m/mm)	80	100	120
Feed rate f (mm/rev)	0.05	0.10	0.15
Cutting depth a (mm)	0.2	0.4	0.6

**Table 2:** L9 Orthogonal Array using the Taguchi Method.

Exp. No	Cutting speed (V)	Feed rate (f)	Cutting depth (a)
1	1	**1**	**1**
2	1	2	2
3	1	3	3
4	2	**1**	3
5	2	2	**1**
6	2	3	**2**
7	3	**1**	2
*8*	3	2	3
*9*	3	3	**1**

These parameters were chosen with the understanding that the cutting speeds needed to machine titanium alloys exceed 54 m/min, with the majority of researchers employing speeds between 80 and 160 m/min. Because titanium alloys are used in small applications, there are small cutting depths within the scope of 0.1: 1 mm in addition to the cutting speed range. These specifications were chosen to make the machining process easier for material that is challenging to cut (TC21 Ti-alloy).

Parameter optimization for a given process is the first step in the Taguchi technique, which improves quality performance and reduces experimental turning costs. Process parameters are optimized by the Taguchi technique, which produces ideal parameters that are not affected by noise or external circumstances. The Taguchi method employs a loss function to measure the deviation between the desired value and the experimental results. A signal-to-noise (S/N) ratio is further converted from this loss function.

The required response category determines the signal-to-noise (S/N) ratio. The signal-to-noise ratio (S/N) can be computed using one of three formulas. Equation (1) indicates that smaller is better (SB), Eq. (2) indicates that nominal is better (NB), and Eq. (3) presents larger is better (LB)^29,30^.

The smaller is the better (minimize):

1 $$S/N = \eta = - 10\log 10\left( {\sum \left( {Y^{2} } \right)/n} \right)$$

Nominal is best

2 $${\text{ S}}/{\text{N}} = \eta { } = 10log10\left( {\frac{{\overline{Y}^{2} }}{{S^{2} }}} \right)$$

Larger is better (maximize)

3 $${\text{S}}/{\text{N}} = \eta = { } - 10log10\left( {\Sigma \left( {\frac{1}{{Y^{2} }}} \right)/n} \right)$$

To verify the precision of the DIAVITE DH-5 tester and scanning electron microscope (SEM), a calibration stage was established using an international standard block. Three tests were conducted to calculate the average response for the surface roughness measurements.

## Results and discussion

### Surface roughness

#### The analysis of ratio of signal to noise

In this study, higher effectiveness is demonstrated by lower surface roughness (Ra) values. For optimizing surface roughness, the "smaller is better" approach was adopted to identify the best machining parameters. The optimal levels of machining parameters are those with the highest signal-to-noise ratio (S/N). Table 3 presents the calculated S/N ratios for each parameter level. The experiment with the highest S/N ratio, experiment no. 9 (V3 f3 a2), was identified as providing the ideal surface roughness parameters, with settings of V = 120 m/min, f = 0.15 mm/rev, and a = 0.4 mm. Table 4 details the best-performing parameters.

**Table 3:** Surface Roughness (Ra) Experimental Results.

Experimental serial number	Speed of cut V **(m/min)**	Feed rate f	Depth of cut a (mm)	Surface roughness **Ra** (μm) Mean	**Ra** S/N (um)
1	SO	0.05	0.2	0.703333	3.0568
2	80	0.10	0.4	0.166667	15.5630
3	80	0.15	0.6	0.690000	3.2230
4	100	0.05	0.4	0.446667	7.0003
5	100	0.10	0.6	0.630000	4.0132
6	100	0.15	0.2	0.193333	14.2739
7	120	0.05	0.6	0.693333	3.1812
S	120	0.10	0.2	0.126667	17.9468
*9*	120	0.15	0.4	0.070000	23.0980

**Table 4:** Optimal Process Parameters.

Exp. No	Process parameters	Surface roughness (μm)
2	V_3_ f_3_ B2	0.07

For each parameter, the average signal-to-noise (S/N) ratio is calculated to rank the cutting parameters by their importance. For instance, Equations (4-6) illustrate the method for calculating Factor V (cutting speed)^31^:

4 $${\text{SNv}}_{{1}} = \left( {{3}.0{568 } + { 15}.{563}0 \, + { 3}.{223}0} \right) \, /{ 3 } = { 7}.{281}$$

5 $${\text{SNv}}_{{2}} = \left( {{7}.000{3 } + { 4}.0{132 } + { 14}.{2739}} \right) \, /{ 3 } = { 8}.{429}$$

6 $${\text{SNv}}_{{3}} = \left( {{3}.{1812 } + { 17}.{9468 } + { 23}.0{98}0} \right) \, /{ 3 } = { 14}.{742}$$

The range is determined in order to calculate the effect of this factor:

7 $$\Delta = {\text{SNV}}_{{{\text{max}}.}} {-}{\text{ SNV}}_{{{\text{min}}.}} = {14}.{742 }{-}{ 7}.{281 } = { 7}.{461}$$

According to Table 5 in this study, the cutting depth parameter has the biggest variation between its levels and, as a result, has a significant impact on the experiment response (surface roughness), while the cutting speed parameter has less of an impact.

**Table 5:** Signal-to-Noise (S/N) Response and Delta Value for Each Parameter.

Level	V	F	A
1	7.281	4.413	11.759
2	S.429	12.50S	15.220
3	14.742	13.532	3.472
Delta	7.461	9.119	11.748
Rank	3	2	1

**Figure 4:**
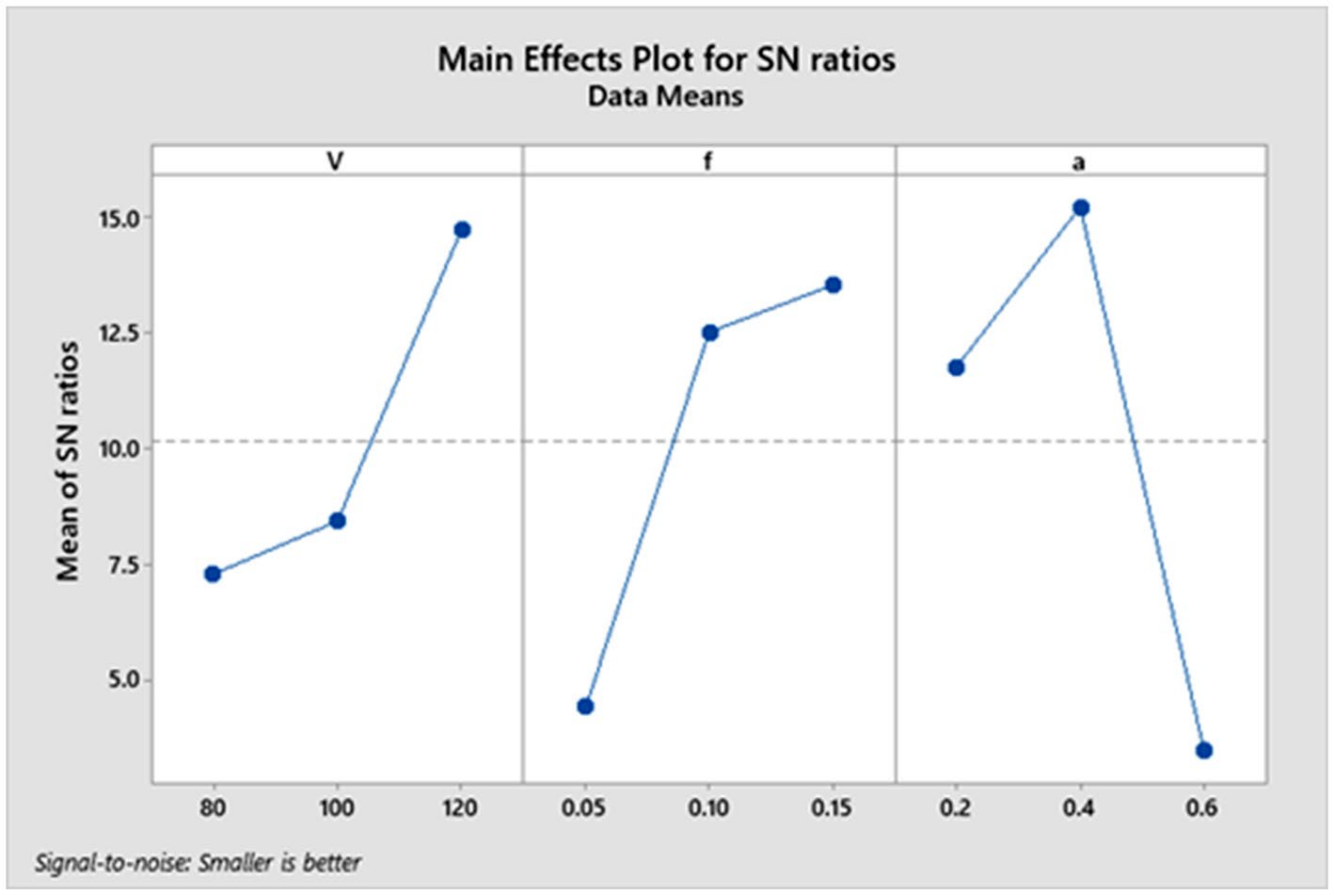
Average Signal-to-Noise (S/N) Ratio for Surface Roughness (Ra).

The variation for all parameters with signal-to-noise ratio is displayed in the following figure. The delta values for parameters V, f, and an are 7.461, 9.119, and 11.748, respectively, as shown in Table 5. Figure 4 plots the delta value for each parameter^32–38^.

**Figure 5:**
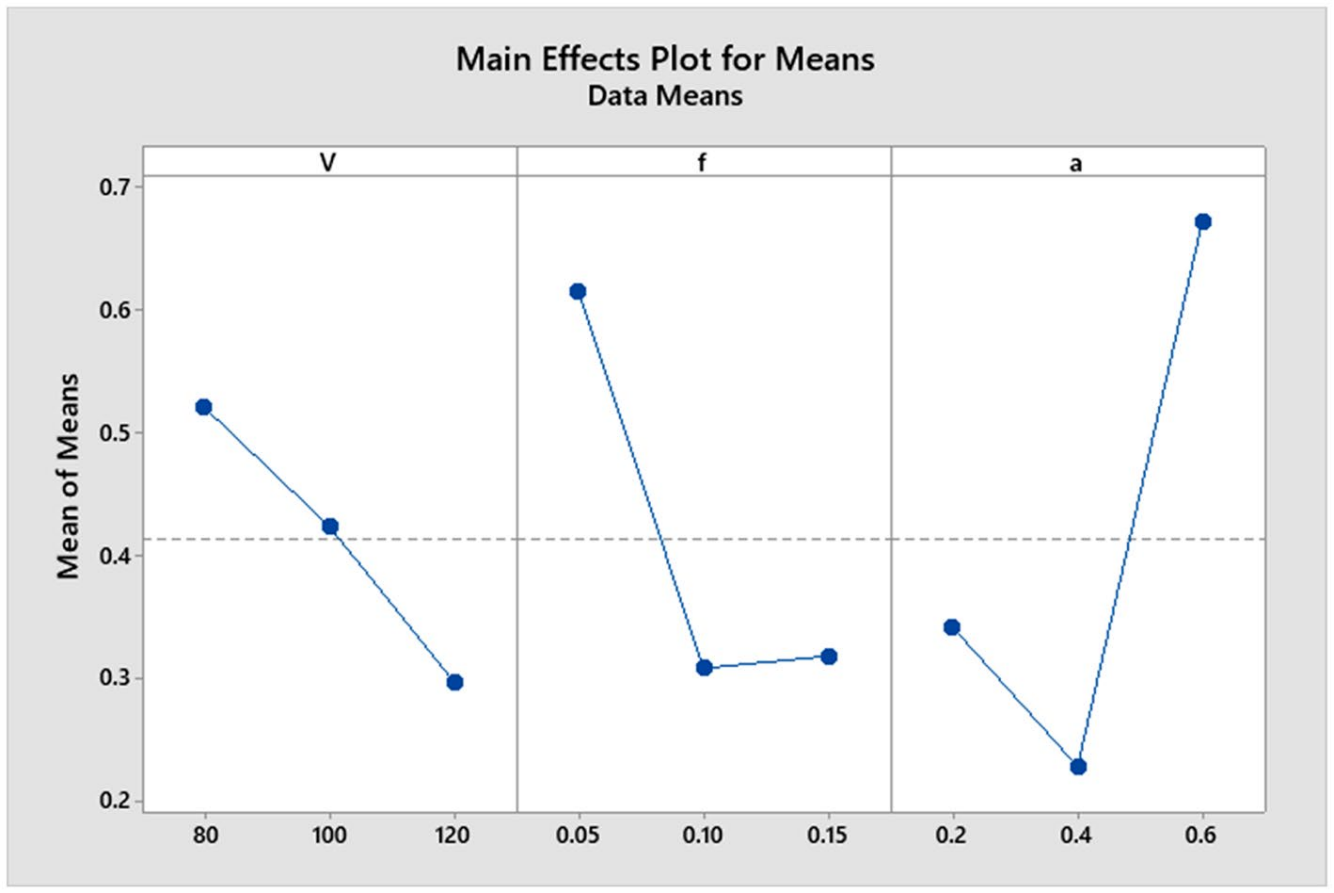
Main effects plot for means for Ra.

From Fig. 5, it is shown that the surface roughness value is in direct relation to the cutting speed parameter that decreases when cutting speed and feed rate increase. Still, it decreases from level one to level two and then increases from level two to level three with increasing depth of cut. It is noticed that there is a long variation between the levels of each of the two parameters (depth of cut and feed rate). Consequently, the mentioned two parameters occupy the first two affected parameters on the machining process. Therefore, the cutting speed is at the last affected parameter in the machining process.

#### Variance Analysis (ANOVA)

ANOVA was employed to assess the effects of cutting depth, feed rate, and speed on surface roughness. The results are presented in Table 6, with a confidence level of 95%. Cutting depth was identified as the most influential factor, contributing 46.6% to the variability in surface roughness. Conversely, cutting speed was the least significant parameter, accounting for only 20% of the variation. All parameters were deemed significant in the machining process, as indicated by their P-values being lower than 0.05.

**Table 6:** ANOVA-Based Optimizations**.**

Source	Degree of freedom (DOF)	The sum ofsquares (SS)	Adj(SS)	Adj(MS)	F	P-Value	Percentcontribution(PC) %	Remarks
V	z	96.84	96.S4	48.419	24.47	0.039	20	Sisnificant
F	2	149.7	149.7	74.865	37.84	0.026	32	Significant
A	**2**	218.7	218.7	109.33	55.25	0.018	46.6	Sisnificant
Residual error	2	3.957	3.957	1.979				
Total	8	469.191						

### Tool wear

#### The analysis of ratio of Signal to noise

Greater effectiveness in this work is indicated by decreased tool wear. To achieve the best machining parameters, the smaller-is-better approach to tool wear was chosen. An ideal level is represented by the machining parameter levels that have the highest signal-to-noise ratio (S/N). Table 7 shows the computed (S/N) ratio for each parameter level. Using the S/N ratios, experiment no. 9 (V3 f. 3 a2), which has the highest S/N ratio, yields the ideal surface roughness parameters. The parameters for this experiment are V = 120 m/min, f = 0.15 mm/rev, and a = 0.4 mm. Table 8 lists the optimal parameters.

**Table 7:** Experimental Results for Tool Insert Wear, Observed and Predicted Signal-to-Noise (S/N) Ratios for Tool Wear.

Experimental serial number	Cutting speed **V** (m/mm)	Feed rate f (mm/rev)	Cutting depth a (mm)	Tool wear **Ymax** Mean (µm)	Ymax S/N (µm)
1	80	0.05	0.2	227.089	− 47.1239
2	80	0.10	0.4	201.712	− 46.0946
3	80	0.15	0.6	232.031	− 47.3109
4	100	0.05	0.4	195.657	− 45.8299
5	100	0.10	0.6	209.539	− 46.4253
*6*	100	0.15	0.2	170.286	− 44.6236
7	120	0.05	0.6	215.970	− 46.6879
8	120	0.10	0.2	143.500	− 43.1370
9	120	0.15	0.4	142.375	− 43.0687

**Table 8:** Optimal parameters of process.

Esp. No	Process parameters	Tool wear (μn)
*9*	**V3 f.** **3** m	142**.375**

For each factor, the average signal-to-noise (S/N) ratio is calculated to prioritize the parameters based on their importance. Equations (8–10) provide a sample calculation for Factor V (cutting speed)^31^:

8 $${\text{SNv}}_{{1}} = \left( { - {47}.{1239 } - { 46}.0{946 } - { 47}.{31}0{9}} \right) \, /{ 3 } = \, - {46}.{84}$$

9 $${\text{SNv}}_{{2}} = \left( { - {45}.{8299 } - { 46}.{4253 } - { 44}.{6236}} \right) \, /{ 3 } = \, - {45}.{63}$$

10 $${\text{SNv}}_{{3}} = \, ( - {46}.{6879 } - { 43}.{137}0 \, - { 43}.0{687}) \, /{ 3 } = \, - {44}.{3}0$$

The range is calculated to assess the impact of this factor:

11 $$\Delta \, = {\text{ SNV}}_{{{\text{max}}.}} {-}{\text{ SNV}}_{{{\text{min}}.}} = \, - {44}.{3}0 \, {-} \, \left( { - {46}.{84}} \right) \, = {2}.{54}$$

According to Table 9, the cutting speed parameter exhibits the largest variation among its levels in this study, leading to a significant impact on the experimental response (tool wear). In contrast, the feed rate parameter shows minimal influence on the same.

**Table 9:** Signal-to-Noise (S/N) Response and Delta Value of Ymax for Each Parameter.

Level	V	F	A
1	− 46.84	− 46.55	− 44.96
2	− 45.63	− 45.22	− 45.00
3	− 44.30	− 45.00	− 46.81
Delta	2.54	1.55	1.85
**Rank**	1	3	2

**Figure 6:**
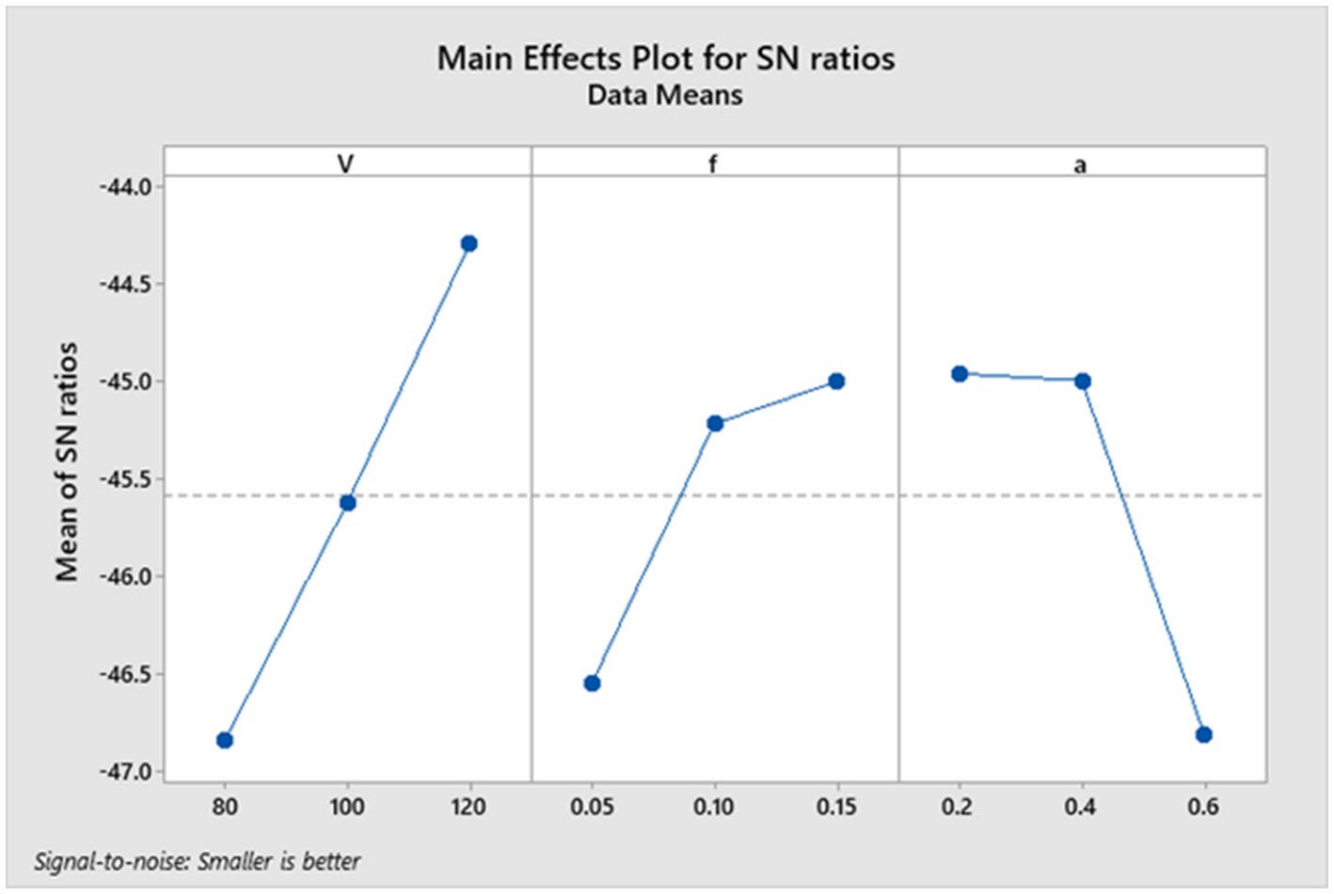
Average Signal-to-Noise (S/N) Ratio for Ymax.

The variation for all parameters with signal-to-noise ratio is displayed in the following figure. The delta values of parameters V, f, and an are 2.54, 1.55, and 1.85, respectively, as indicated by Table 9. Figure 6 plots the delta value for every parameter^32–39^.

**Figure 7:**
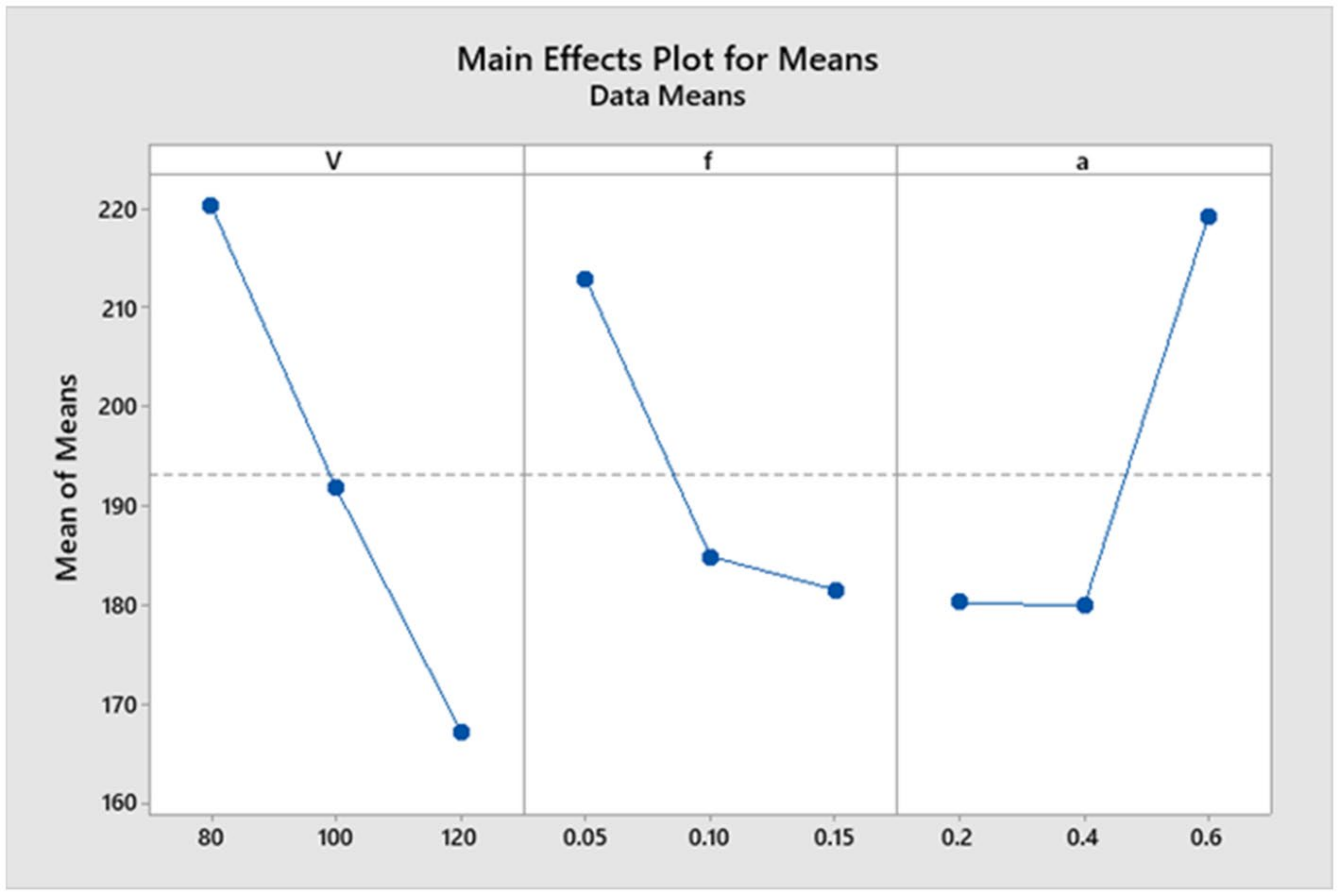
Main effects plot for Means.

From Fig. 7, it is shown that the tool wear value decreases with an increase in the cutting speed parameter and feed rate. Still, it decreases from level one to level two and then increases from level two to level three with increasing depth of cut. It is noticed that there is a long variation between the levels of each of the two parameters (cutting speed and depth of cut). Consequently, the two parameters occupy the first two affected parameters in the machining process. Therefore, the feed rate is the last affected parameter in the machining process.

#### Variance analysis (ANOVA)

ANOVA was applied to assess the impact of feed rate, cutting depth, and cutting speed on tool wear. The results, detailed in Table 10, are presented with a 95% confidence level. The findings indicate that cutting speed is the most significant parameter, contributing 46.7% to tool insert wear. On the other hand, the feed rate is the least influential, accounting for a 32.1% contribution.

**Table 10:** ANOVA-Based Optimization for Tool Wear.

Source	Degree of freedom (DOF)	The sum of squares (SS)	Adj (SS)	Adj (MS)	F	P-Value	Percent contribution (PC)%	Remarks
V	**2**	9.7240	9.7240	4.8620	43.55	0.022	46.7	Significant
F	**2**	4.2024	4.2024	2.1012	18.82	0.050	20.2	Significant
A	**2**	6.6882	6.6882	3.3441	29.95	0.032	32.1	Significant
Residual error	2	0.2233	0.2233	0.1116				
Total	8	20.8379						

#### The mechanisms of Tool wear

**Figure 8:**
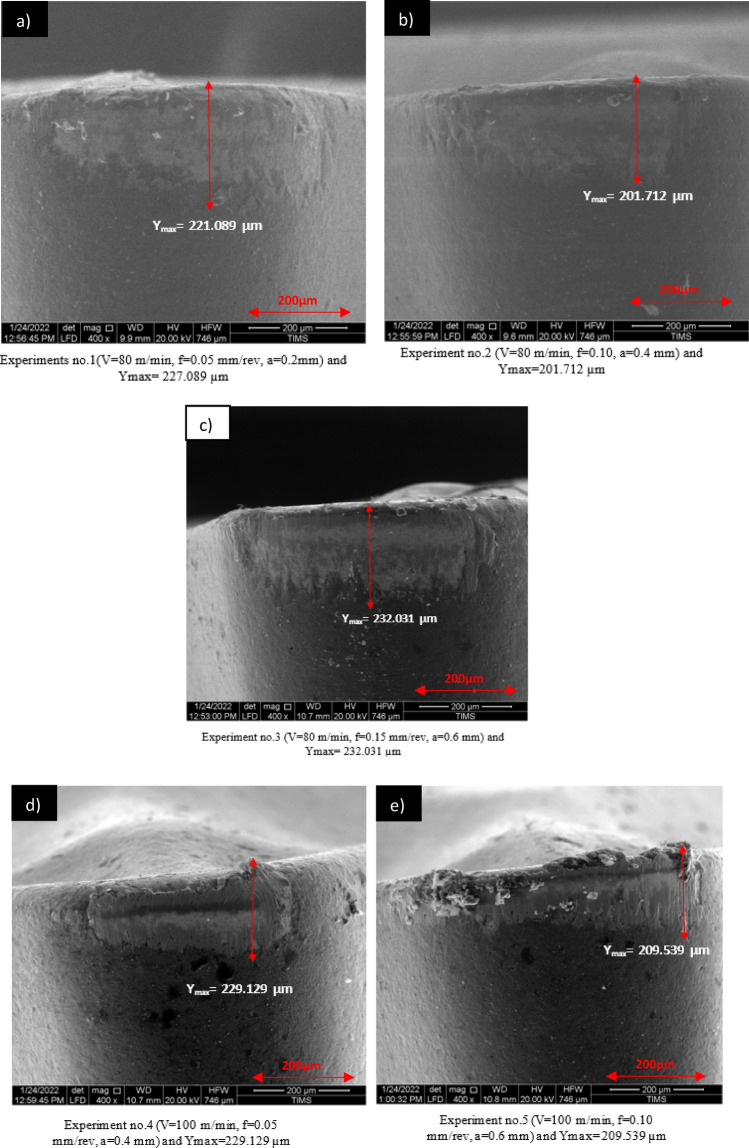

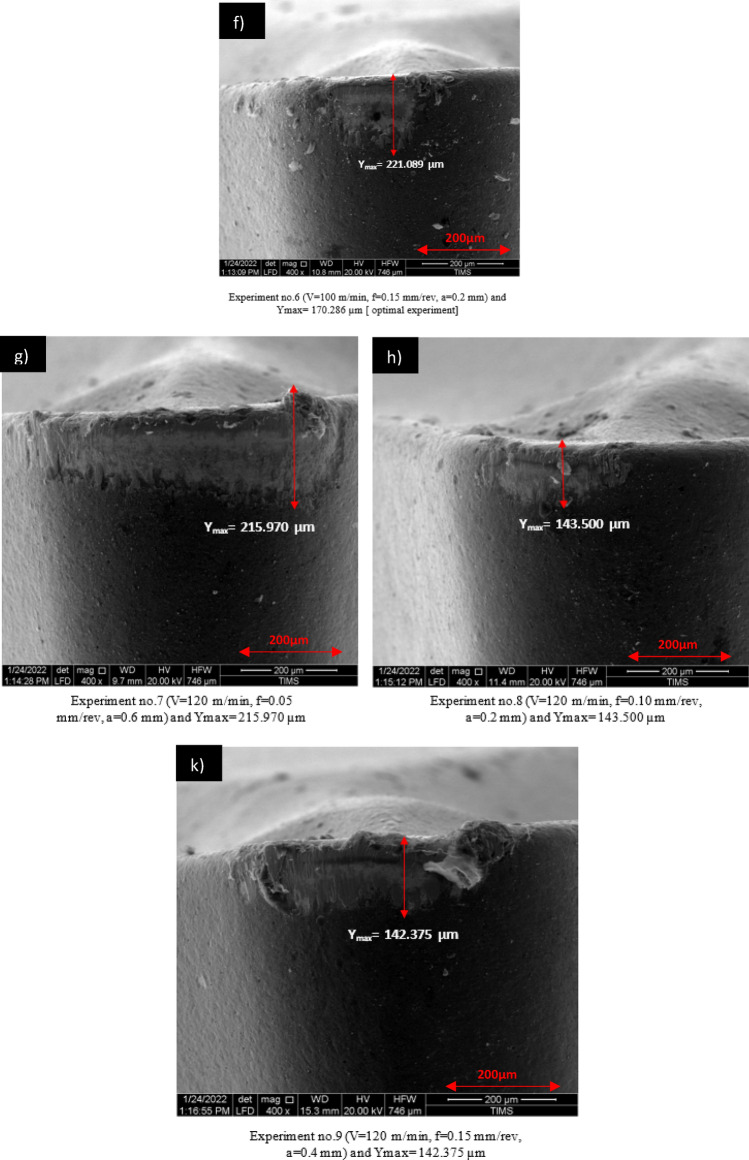
SEM images of the wear of flank face.

The wear height of the tool for each experiment was measured using a scanning electron microscope (SEM) to optimize through the Taguchi method. This approach was utilized to analyze the complexity of tool wear during the turning of TC21, as detailed in Table 4. The scanned images from each experiment are displayed in Fig. 8. Among the parameters tested, cutting speed was found to have the most significant impact on tool wear during the turning experiments with TC21.

### Comparison of the results between the initial and heat treated specimens

**Figure 9:**
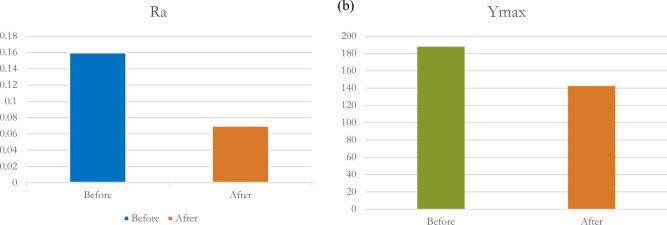
(**a**) Surface roughness improvement. (**b**) Tool wear improvement.

After accomplishing the trials for two stages (as received and as treated), it was noticed that the improvement of machinability after heat treatment. The surface roughness decreased from 0.16 to 0.07 µm with an improvement percentage of 56.25%, and the tool wear from 187.77 to 142.375 µm with an improvement percentage of 24.18%. The improvement will be shown in Fig. 9(a) and (b).

Although the increase in hardness after heat treatment from 34.8 to 50.33 HRC, the refinement of grain size in microstructure from (α + β) to (α + β) with secondary α platelets (αs) formed in residual β matrix as a result of forming secondary alpha platelets in beta matrix that leads to the increased volume fraction of beta phase as shown in Fig. 2(a,b) in section "Material and method", and by choosing an appropriate cutting tool insert (coated carbide) and select optimized cutting parameters, all these factors lead to improve the machinability after heat treatment.

Table 11 displays the comparison of the results between as-received and heat-treated samples. It is noticed from Table 11 the improvement of the two responses after applying the heat treatment process. The following bar charts as shown in Fig. 9 show the improvement that occurred after heat treatment in both responses (Surface roughness and tool wear).

**Table 11:** Comparison of the results between as-received and heat-treated samples.

Results	Surface Roughness (Ra)		Tool wear (Y_max_)	
	As received	As treated	As received	As treated
Mean	0.16 µm^28^	0.07 µm	187.77 µm^28^	142.375 µm
S/N Ratio	15.917 µm	23.0983 µm	− 45.4725 µm	− 43.0687 µm
Prediction model	6.564%	8.76%	8.76%	7.47%
Improvement percentage	56.25%		24.18%	

## Conclusions

The conclusions drawn from the surface roughness prediction model and the tool wear prediction model for TC21 Ti-alloy tool inserts are as follows:

1. Under optimal conditions, experiment no. 9 achieved the lowest surface roughness with the highest signal-to-noise ratio. The best machining parameters were V = 120 m/min, f = 0.15 mm/rev, and a = 0.4 mm, achieving a Ra of 0.07 µm.
2. Cutting depth is the most influential parameter on surface roughness, accounting for 46.6% of its variability.
3. Cutting speed has the least impact on surface roughness, contributing 20% to its variation.
4. For tool wear, experiment no. 9 also displayed the lowest levels under optimal conditions, again with the highest signal-to-noise ratio. The optimal machining parameters were the same as above, with a resultant tool wear measurement of Y = 142.375 µm.
5. The speed of cut is the most significant factor affecting tool insert wear, with a 46.7% contribution.
6. The feed rate, contributing 20.2% to the overall tool wear, is the least significant parameter.

In future work, we can consider other machining parameters besides the mentioned parameters. The considered machining parameters may be the consuming time, machining cost, the variety of cutting tool inserts, and cutting tool geometries (rake and clearance angle). Also, there are other things to be considered like machining conditions (dry, minimum quantity lubrication, nitrogen cooling, etc.) and achieving multi-heat treatment stages with temperature variations, and cooling rate (Furnace cooling, air cooling, and water quenching).

## Data Availability

All data generated or analyzed during this study are included in this published article.
